# Increased hippocampal shape asymmetry and volumetric ventricular asymmetry in autism spectrum disorder

**DOI:** 10.1016/j.nicl.2020.102207

**Published:** 2020-02-05

**Authors:** Rose Richards, Ellen Greimel, Dorit Kliemann, Inga K. Koerte, Gerd Schulte-Körne, Martin Reuter, Christian Wachinger

**Affiliations:** a Department of Child and Adolescent Psychiatry, Psychosomatic and Psychotherapy, University Hospital, Ludwig-Maximilian-University, Nussbaumstr. 5a, 80336 Munich, Germany; b Division of Humanities and Social Sciences, California Institute of Technology, Pasadena, CA 91125, USA; c Department of Psychiatry, Brigham and Women's Hospital, Harvard Medical School, Boston, MA, USA; d A.A. Martinos Center for Biomedical Imaging, Massachusetts General Hospital, 149 Thirteenth Street, Suite 2301, Charlestown, MA, USA; e Department of Radiology, Harvard Medical School, Boston, Massachusetts, USA; f Image Analysis, German Center for Neurodegenerative Diseases (DZNE), Bonn, Germany

**Keywords:** Autism, Asymmetry, MRI, Amygdala, Hippocampus, Ventricles

## Abstract

Autism spectrum disorder (ASD) is a prevalent and fast-growing pervasive neurodevelopmental disorder worldwide. Despite the increasing prevalence of ASD and the breadth of research conducted on the disorder, a conclusive etiology has yet to be established and controversy still exists surrounding the anatomical abnormalities in ASD. In particular, structural asymmetries have seldom been investigated in ASD, especially in subcortical regions. Additionally, the majority of studies for identifying structural biomarkers associated with ASD have focused on small sample sizes. Therefore, the present study utilizes a large-scale, multi-site database to investigate asymmetries in the amygdala, hippocampus, and lateral ventricles, given the potential involvement of these regions in ASD. Contrary to prior work, we are not only computing volumetric asymmetries, but also shape asymmetries, using a new measure of asymmetry based on spectral shape descriptors. This measure represents the magnitude of the asymmetry and therefore captures both directional and undirectional asymmetry. The asymmetry analysis is conducted on 437 individuals with ASD and 511 healthy controls using T1-weighted MRI scans from the Autism Brain Imaging Data Exchange (ABIDE) database. Results reveal significant asymmetries in the hippocampus and the ventricles, but not in the amygdala, in individuals with ASD. We observe a significant increase in shape asymmetry in the hippocampus, as well as increased volumetric asymmetry in the lateral ventricles in individuals with ASD. Asymmetries in these regions have not previously been reported, likely due to the different characterization of neuroanatomical asymmetry and smaller sample sizes used in previous studies. Given that these results were demonstrated in a large cohort, such asymmetries may be worthy of consideration in the development of neurodiagnostic classification tools for ASD.

## Highlights

• Found increased subcortical asymmetry associated with autism.
• Utilized a new measure of shape asymmetry for analysis of structural differences.
• Observed significantly increased shape asymmetry of the hippocampus.
• Observed significantly increased volumetric asymmetry in the lateral ventricles.
• Focalized abnormalities may result in detectable shape (but not volume) differences.

## 1 Introduction

Autism spectrum disorder (ASD) is a pervasive neurodevelopmental disorder that begins in early childhood and persists throughout life. ASD is characterized by impairments in communication and social interaction, along with the exhibition of stereotyped repetitive patterns of behavior and restricted interests (DSM-V, American Psychiatric Association, 2013). Prevalence estimates continue to suggest that ASD is one of the most prevalent developmental disorders (CDC 2014; Brownsell et al., 2011; Elsabbagh et al., 2012; Rice et al., 2012), estimated to occur in approximately one in every 160 individuals worldwide (World Health Organization, 2013). Despite the prevalence of the disorder, however, research has not yet been able to determine a conclusive etiology (Hughes, 2008; Pendergrass et al., 2014).

In recent years, extensive research utilizing magnetic resonance imaging (MRI) has been conducted to investigate structural and functional differences found in ASD, and various neuroanatomical differences have been proposed as potential biomarkers for the disorder (for a recent review, see Li et al., 2017). Although MRI has enabled researchers to search for neuroanatomical biomarkers and attempt to develop MRI-based classification algorithms and computer-aided diagnostic systems to facilitate diagnosis (Dekhil et al., 2017; Ecker et al., 2010; Ismail, 2016; Mateos-Perez et al., 2018; Mostapha et al., 2015; Nielsen et al., 2012; Plitt et al., 2014), findings of potential structural biomarkers for ASD have been mixed and partly inconclusive.

The amygdala has consistently been a structure of interest in the search for a neuropathological classification of autism, given its important role in emotional and social functions that are impaired in individuals with ASD (Baron-Cohen et al., 2000; Brothers, 1990; Howard et al., 2000). The hippocampus is frequently investigated in tandem with the amygdala, due to its connection to the amygdala within the limbic system and its involvement in core functions in the “social brain” (Crespi and Badcock, 2008; Goodman et al., 2014; Groen et al., 2010; Hughes, 2008). Despite that both structures have been studied in individuals with ASD, research has demonstrated inconsistent evidence for structural differences in these regions. While many researchers have found increased volume in the amygdala (Groen et al., 2010; Howard et al., 2000; Kim et al., 2010; Munson et al., 2006; Murphy et al., 2012; Nordahl et al., 2012; Sparks et al., 2002; Schumann et al., 2004) others have found no evidence for this increase (Haar et al., 2014) and one study even found significantly decreased volume within the amygdala (Aylward et al., 1999). Similarly, while some studies have found increased volume in the hippocampus (Groen et al., 2010; Murphy et al., 2012, Rojas et al., 2006), others have found no significant differences in hippocampal volume (Aylward et al., 1999; Piven et al., 1998) and some have found significantly decreased volume within the hippocampus (Eilam-Stock et al., 2016; Nicolson et al., 2006; Schumann et al., 2004; Sparks et al., 2002).

Ventricles have also gained much attention in ASD research, given that morphometric differences such as lesions and hypertrophy of neighboring brain volume (Carper et al., 2002; DeLong and Bauman, 1987; Herbert, 2005; Vidal et al., 2008) have been found in ASD, and such issues of hemispheric development are likely to exert influence on the shape and size of the lateral ventricles (Geschwind and Galaburda, 1985). The majority of studies have found greater ventricular volumes associated with ASD (Filipek et al., 1992; Howard et al., 2000; Lange et al., 2015; McAlonan et al., 2002; Movsas et al., 2013; Palmen et al., 2005; Piven et al., 1995; Turner et al., 2016; Wolfe et al., 2015) and longitudinal research has also recently identified ventricular enlargement as a risk factor for ASD in a low birth weight population (Movsas et al., 2013). However, some studies have not found evidence for increased ventricular volumes (Creasey et al., 1986; Garber et al., 1989; Hardan et al., 2001; Jacobson et al., 1988), and one study even found reductions in ventricular volume in the right and left frontal and occipital horns (Vidal et al., 2008). Although several studies have investigated volumetric differences in the ventricles, little attention has been given to asymmetry of the lateral ventricles and the neighboring subcortical structures in ASD.

Although recent research has linked asymmetric brain alterations to several psychiatric disorders, such as anorexia nervosa (Titova et al., 2013) and schizophrenia (Oertel-Knochel et al., 2012), clear asymmetry patterns for ASD have not yet been established. While Herbert et al. (2005) found ASD-related asymmetry differences in the higher-order association cortex and Haznedar et al. (2006) found a reversal of typical hemispheric asymmetry in ASD patients, these results were observed in small samples (*N* = 16 and *N* = 15, respectively) and have yet to be replicated in larger samples. Atypical diffusion tensor hemispheric asymmetry has been observed within the superior temporal gyrus and temporal stem of individuals with ASD (Lange et al., 2010), however, these results have also yet to be replicated in a large sample. Dougherty et al. (2016) recently found evidence for abnormal asymmetry of the fusiform gyrus associated with symptom severity in a large sample of males with ASD, however, aside from these studies, asymmetry research on structures thought to underlie impaired processes in ASD is quite sparse. Recently, Monterrey et al. (2017) reported incidental findings of hippocampal asymmetry in twin pairs with ASD (*N* = 15), as well as ventricular abnormalities (i.e. either enlargement or asymmetry), which occurred more frequently in twin pairs with ASD (*N* = 18 pairs) than in control twin pairs (*N* = 10 pairs), however, these findings have not yet been replicated in larger samples. While relationships have been found between other psychiatric disorders and abnormal asymmetries of subcortical structures, such as the amygdala in schizophrenia (Niu et al., 2004), the hippocampus in depression (Xia et al., 2004), and both the hippocampus and the amygdala in Alzheimer's disease (Wachinger et al., 2016), asymmetry patterns within these subcortical structures have not yet been established for ASD.

It is important to note that the majority of studies conducted on structural abnormalities in ASD, especially studies investigating subcortical structures, have relied on small sample sizes. Although the recent advent of the Autism Brain Imaging Data Exchange (ABIDE) database has permitted research using a larger number of subjects, few studies have utilized the ABIDE sample to investigate potential ASD-related differences in subcortical regions (Cerliani et al., 2015; Plitt et al., 2014; Turner et al., 2016). The ABIDE dataset has received some criticism due to its breadth across various sites utilizing different scanners, given the inherent confounding effects of scanner variations (Auzias et al., 2015), however, research investigating such confounding scanner effects has demonstrated that robust results may still be obtained across images obtained from different scanners in multisite analyses (Noble et al., 2017).

Additionally, ABIDE presents the unique opportunity to include a large sample of females within analyses. Research including females with ASD is limited, likely in part due to the differential prevalence rates among males and females (Fombonne, 2009), but also due to the fact that several studies on ASD have excluded females from analyses altogether. Therefore, the present study will include females in the analyses, given that a large sample is available in the ABIDE dataset.

Given the scarcity of asymmetry research and the problems inherent to findings in small ASD samples, we therefore aim to investigate ASD-related asymmetry within subcortical structures, namely, the amygdala, hippocampus, and lateral ventricles within a large sample of ASD patients and healthy controls. The development of hemispheric asymmetry is noted to underlie important functions, such as visuospatial processing (Zhen et al., 2017) and language (Vigneau et al. 2006), and although many asymmetries are known to emerge throughout normal development, others emerge as a result of disordered hemispheric development, such as disturbed neuronal migration or the emergence of atrophic lesions (Geschwind and Galaburda, 1985). Such disturbances in neuronal development have been hypothesized to underlie various learning disabilities and psychiatric conditions (Geschwind and Galaburda, 1985). Although asymmetries within subcortical structures have previously been linked to an increased susceptibility to cognitive and psychiatric disorders (for a detailed review and large-scale asymmetry study, see Guadalupe et al., 2016), little is known about the relationship between subcortical asymmetries and ASD. Thus, we analyze asymmetries of contralateral brain structures that have been previously implicated in ASD, as they present a unique, subject-specific reference element for comparison and can potentially serve as a personalized marker of the disorder. Concurrently to our work, Postema et al. (2019) have also investigated subcortical volume asymmetries in ASD.

While most existing asymmetry analyses utilize purely volumetric measures of asymmetry, we aim to analyze both volumetric and shape asymmetries in the present study, given that clear asymmetry patterns for these structures have not yet been established for ASD. In this article, we will work with a new measure of structural brain asymmetry that has previously been used to study Alzheimer's disease (Wachinger et al., 2016; Wachinger et al., 2018). This method operates on spectral shape descriptors in the *BrainPrint* (Wachinger et al., 2015), which describe the geometry of a brain structure with a high-dimensional vector. Hence, it has the potential to be more sensitive to anatomical variations than commonly used volume measurements. This shape asymmetry measure captures the magnitude of the asymmetry and therefore combines directional and undirectional asymmetry. Directional asymmetry refers to hemispheric differences that systematically show a stronger effect on one of the hemispheres, e.g., larger changes on the right than on the left. In contrast, undirectional asymmetry does not have a consistent hemispheric effect. Alternative approaches, such as voxel-wise techniques or statistical shape models compute statistics across the population and are well suited for measuring directional asymmetry, but they cannot detect undirectional asymmetry. In addition, these techniques only allow for a qualitative assessment of shape asymmetry, but do not provide quantitative measure of shape asymmetry. The sensitive representation of geometry together with ability to identify undirectional asymmetry are particularly promising for ASD, given the complex and multi-faceted nature of the disorder, which may be accompanied by subtle focalized differences in morphology that are difficult to detect using purely volumetric measures.

## 2 Method

### 2.1 MR image acquisition

Structural MRI data were obtained from the ABIDE 1 database of preprocessed MR images (Craddock et al., 2013; http://preprocessed-connectomes-project.org/abide/). The ABIDE database contains MRI data for 539 individuals with ASD and 573 healthy controls, aged 6–65 years, collected at 17 international sites. All sites acquired MRI data using 3-Tesla scanners with T1-weighted scans with isotropic voxels (1 × 1 × 1-mm resolution). All data were obtained with informed consent, approved by institutional review boards (IRBs) at their respective collection sites, and were fully anonymized in accordance with HIPAA regulations. Data were also visually inspected and assigned quality ratings from three independent raters prior to online distribution. Further information on data acquisition and site-specific details (i.e., protocols, test batteries used, and scanning parameters) is available at http://fcon_1000.projects.nitrc.org/indi/abide/abide_I.html

### 2.2 Sample selection

Following inspection of the demographics of the ABIDE dataset, subjects older than 35 (*N* = 21) were removed both due to the relatively low number of subjects aged older than 35 within the dataset, and to lessen the potential confounding effects of normal brain aging experienced during mid- and late-life stages on our results. Only individuals with full-scale IQ (FSIQ) scores within two standard deviations of the overall ABIDE sample mean were included in the final sample to reduce the potential influence of outlying FSIQ scores. Finally, quality ratings provided by ABIDE were then used to remove subjects with scans rated as “poor” quality from the dataset. These quality ratings were obtained following manual inspection of the raw images in the ABIDE dataset by three independent reviewers. In cases where any rater disagreement occurred or where low ratings were given, images were visually inspected by the authors to ensure that segmentation was performed successfully before including these scans in analyses. Visual inspection resulted in the removal of two participants with ASD and two control participants with poor quality scans, which prevented the calculation of intracranial volume. This yielded data for a total of 948 individuals (ASD = 437, Controls = 511) from 17 different sites.

### 2.3 Subjects

The present study used a sample of 437 ASD patients (382 males), with ages ranging from seven to 34.6 (*M* = 15.95, *SD*=6.17), and 511 healthy controls (419 males), with ages ranging from 6.47 to 34.1 (*M* = 16.13, *SD* = 6.20). The two groups did not differ significantly in age (*t* = 0.43, *p* = 0.66) nor in variances in age (*F* = 0.99, *p* = 0.45). Our ASD sample is comprised of individuals diagnosed with all formerly used DSM-IV-TR subtypes for ASD (now classified together under ASD in the DSM-V; APA 2013), please see Table 1 for further detailed demographic information and summary statistics.

**Table 1:** Demographic information and summary statistics by diagnosis.

	Autism spectrum disorder	Healthy controls
*N*	437	511
Sex (M/F)	382/55	419/92
% (M/F)	87.4/12.5	82.0/18.0
Age (years)	15.95 ± 6.17	16.12 ± 6.20
FSIQ Mean	106.38 ± 14.18	111.07 ± 11.74
Intracranial Volume (mm^3^)	1336,921 ± 272,115.55	1322,270 ± 254,188.55
Volumetric Amygdala Asymmetry	185.21 ± 164.95	168.9 ± 141.56
Amygdala Shape Asymmetry	2.13 ± 1.29	2.07 ± 1.14
Volumetric Hippocampal Asymmetry	285.05 ± 398.59	259.35 ± 351.15
Hippocampal Shape Asymmetry	2.15 ± 1.64	1.91 ± 1.14
Volumetric Ventricular Asymmetry	1637.52 ± 1843.34	1300.51 ± 1403.56
Ventricular Shape Asymmetry	3.76 ± 2.29	3.87 ± 2.34

Demographic information and summary statistics by diagnosis. Note: the ASD group is comprised of individuals diagnosed with all formerly used DSM-IV-TR subtypes, including 289 (66.1%) individuals diagnosed with Autism, 73 (16.7%) individuals diagnosed with Asperger's, 25 (5.7%) individuals diagnosed with Pervasive Development Disorder, and three (0.7%) individuals diagnosed as ASD-Not Otherwise Specified. DSM-IV-TR subtype information was not provided for 47 (11%) of ASD patients. Asymmetry values are non-directional.

All ASD patients and healthy controls had mean full-scale IQ (FSIQ) scores ≥79. Patients FSIQ scores ranged from 79 to 137 (*M* = 106.38, *SD* = 14.18). Healthy controls FSIQ scores ranged from 79 to 138 (*M* = 111.07, *SD* = 11.74). Despite that the two subject groups had similar FSIQ ranges, the variances of the two groups were significantly different (*F* = 1.46, *p* < 0.0001), and control subjects’ FSIQ scores were significantly higher than those of ASD patients, *t*(848) = 5.23*, p* *<* 0*.*0001. No significant difference in intracranial volume was found between ASD patients and controls.

### 2.4 Image analysis

**Fig. 1:**
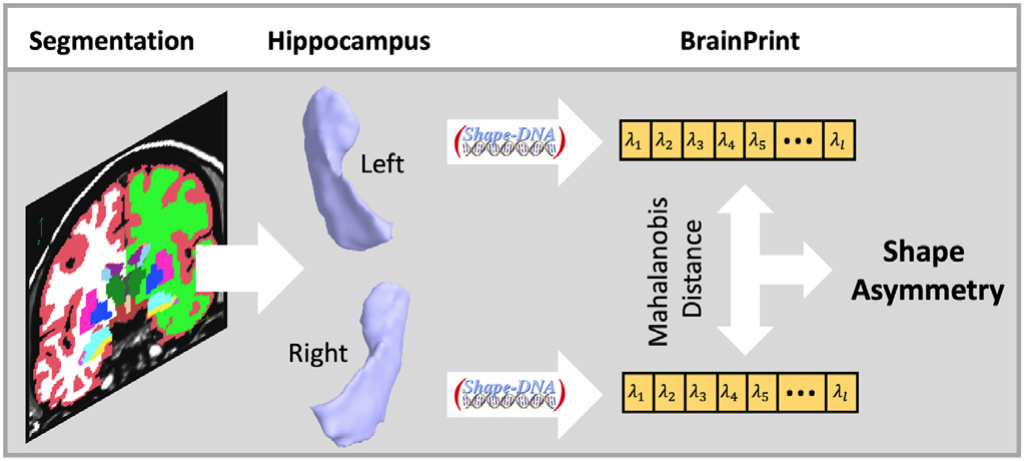
Graphical overview of steps for computing the brain asymmetry with the BrainPrint. MRI scans are segmented with FreeSurfer and meshes for the lateralized structure of interest, here hippocampus, are created. The computation of the shapeDNA results in the spectral shape descriptor in BrainPrint. The Mahalanobis distance between shape vectors yields measure of shape asymmetry.

A graphical overview of the image analysis steps for computing the brain asymmetry with the *BrainPrint* is presented in Fig. 1. The *BrainPrint* (Wachinger et al., 2015) description is based on the automated segmentation of anatomical brain structures with FreeSurfer (Dale and Sereno 1993; Dale et al., 1999; Fischl et al., 1999a, Fischl et al., 1999b; Fischl et al., 2002). We accessed files from the FreeSurfer v5.1 output of the ABIDE Preprocessed initiative (http://preprocessed-connectomes-project.org/abide/index.html). After image segmentation, geometric representations (surface and volumetric meshes) are extracted for the cortical and subcortical structures via the marching cubes algorithm. Marching cubes is a standard algorithm for extracting meshes from voxel maps, where we used the implementation from the FreeSurfer package. Each segmented brain structure is then represented by a mesh, and based on that mesh, a shape descriptor is computed. The shape descriptor transforms the complex geometric representation in a vector, which is easier to work with in the follow-up analyses. It is important that the descriptor captures all the relevant shape information. We use *shapeDNA* (Reuter et al., 2006) as shape descriptor, which performed among the best in a comparison of methods for non-rigid 3D shape retrieval (Lian et al., 2013). *shapeDNA* is based on the eigenvalues of the Laplace-Beltrami operator and, therefore, is isometry invariant (including rigid motion and reflections). Prior applications of shapeDNA and the Laplace-Beltrami operator in medical image analysis are discussed in (Wachinger et al., 2015). Eigenvalues of the Laplace-Beltrami operator Δ can be computed via finite element analysis by solving the Laplacian eigenvalue problem (Helmholtz equation) on the given shape:

Δf=−λf.

The solution consists of eigenvalue $\lambda_{i}\in R$ and eigenfunction *f_i_* pairs, sorted by eigenvalues, $0\leq \lambda_{1}\leq \lambda_{2}\leq \ldots$ (a positive diverging sequence). The first *l* non-zero eigenvalues are computed using the finite element methods and form the *shapeDNA*: $\bar{\lambda }=\left(\lambda_{1},\ldots ,\lambda_{l}\right)$, where we set l=30 in this study. To achieve scale independence, we normalize the eigenvalues:

$$\lambda^{\prime }=\text{vo}l^{\frac{2}{D}}\lambda ,$$

where vol is the Riemannian volume of the *D*-dimensional manifold (Reuter et al., 2006, Wolfe et al., 2015), i.e., the surface area for 2D manifolds, or the volume for 3D solids.

A key property of the eigenvalues is their isometry invariance, i.e., length-preserving deformations will not change the spectrum. Isometry invariance includes rigid body motion as well as reflections, and, therefore, permits the comparison of shapes across individuals or hemispheres by directly comparing the *shapeDNA* without any complex and potentially error-prone image or geometry registration. The *BrainPrint* consists of the spectra for subcortical structures on the 2D boundary surfaces (triangle meshes) and for cortical structures on the full 3D solid (Wachinger et al., 2015). The BrainPrint is very stable with respect to re-meshing and mesh density, as long as the meshes are dense enough to represent the underlying geometry, since the Laplace-Beltrami operator is designed to be independent of the mesh, as opposed to graph Laplace operators (Reuter et al., 2006).

### 2.5 Brain asymmetry from *BrainPrint*

Based on the *BrainPrint*, we measure the asymmetry of the amygdala, hippocampus, and lateral ventricles. Since *shapeDNA* is invariant to reflections, we can directly compute the Mahalanobis distance between the descriptors of a lateralized brain structure, *s*

$$Y_{s}={∥{\bar{\lambda }}_{s}^{\text{left}}-{\bar{\lambda }}_{s}^{\text{right}}∥}_{\Sigma },$$

where we use a diagonal covariance matrix Σ with the *i*th element $\Sigma_{ii}=i^{2}$, for 1 ≤ *i* ≤ *l*, to reduce the impact of higher eigenvalues on the distance (Wachinger et al., 2015). The asymmetry measure is computed independently per subject and therefore presents a within-subject measure; it represents directional and undirectional asymmetry, but does not differentiate between the two types of asymmetry. The approach completely avoids lateral processing bias as it works on both hemispheres independently. Due to the pose invariance of spectral shape descriptors we can directly measure shape asymmetry by computing the distance in a symmetric fashion. Guaranteeing symmetric processing can be rather involved for most other shape representations that first require the construction of local correspondences, where choosing a target hemisphere for registration can potentially bias subsequent analyses. To the best of our knowledge, there is no alternative measure of shape asymmetry.

### 2.6 Statistical analyses

Data were analyzed using linear regression models. Amygdalar, hippocampal, and ventricular asymmetry values were investigated as dependent variables in separate models. Diagnosis group and sex were set as independent variables. Binary classification was used for diagnostic grouping, such that all subjects with a diagnosis of ASD were included together in analyses, regardless of their DSM-IV-TR subtypes. Age, scanning site, intracranial volume, and FSIQ were included as covariates. We evaluated the inclusion of a quadratic age term to the model to account for non-linear aging related effects. However, the likelihood ratio test between linear and quadratic models was not significant, so that we used a linear age term in the final model. All analyses were conducted using R version 3.2.2 for 64-bit Windows (R Core Team, 2015) and RStudio version 0.99.486 for Windows (Rstudio, 2015).

## 3 Results

**Fig. 2:**
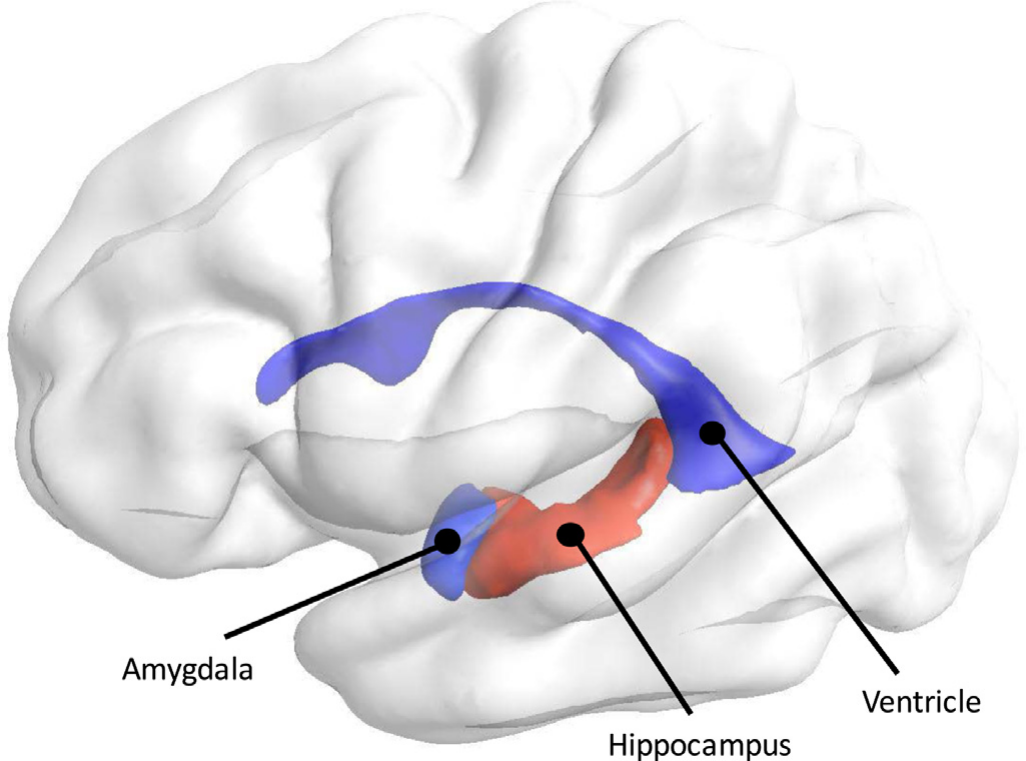
Illustration of brain structures investigated in the present study. Coloring is according to significance of shape asymmetry with respect to diagnosis, where red indicates significant and blue indicates no significant group-related differences in asymmetry. (For interpretation of the references to color in this figure legend, the reader is referred to the web version of this article.)

Multiple regression analyses were calculated to predict volumetric and shape asymmetries in the amygdala, hippocampus, and lateral ventricles based on diagnosis and sex, while controlling for age, scanning site, intracranial volume, and FSIQ. A summary of regression results and the predictive values (*β*) of diagnosis for each of the structural regions can be seen in Table 2. We use Bonferroni correction to account for multiple comparisons and to adjust the significance threshold accordingly. A graphical illustration of regions investigated in the present study, depicted by their significance level of their differences in shape asymmetry, can be seen in Fig. 2.

**Table 2:** Regression results and predictive values of diagnosis, sex, age, ICV, and FIQ on asymmetry.

Region (Asymmetry Type)	*Model*			*Diagnosis*			*Sex*			*Age*			*ICV*			*FIQ*		
	*R^2^*	*F*	*p*	$\hat{\beta }$	*t*	*p*	$\hat{\beta }$	*t*	*p*	$\hat{\beta }$	*t*	*p*	$\hat{\beta }$	*t*	*p*	$\hat{\beta }$	*t*	*p*
Amygdala (volumetric)	**0.05**	**3.39**	**<0.0001**	−19.25	−1.93	0.05	−19.84	−1.40	0.16	0.44	0.41	0.68	−0.01	−1.61	0.11	0.38	0.99	0.33
Amygdala (shape)	**0.16**	**8.72**	**<0.0001**	−0.02	−0.27	0.79	**−0.29**	**−2.72**	**0.006**	−0.01	−0.33	0.74	**−0.01**	**−5.01**	**<0.0001**	−0.01	−2.12	0.03
Hippocampus (volumetric)	**0.04**	**2.59**	**<0.0001**	−17.95	−0.73	0.47	**−115.80**	**−3.32**	**0.0008**	−0.09	−0.03	0.97	0.01	−2.13	0.03	−0.33	−0.35	0.73
Hippocampus (shape)	**0.10**	**5.48**	**<0.0001**	**−0.23**	**−2.66**	**0.008**	**−0.41**	**−3.22**	**0.001**	0.01	−0.011	0.99	**0.01**	**−4.71**	**<0.0001**	0.01	−0.16	0.87
Ventricles (volumetric)	**0.06**	**3.66**	**<0.0001**	**−321.41**	**−3.04**	**0.002**	139.12	0.93	0.36	25.57	2.28	0.02	**0.01**	**6.63**	**<0.0001**	0.36	0.09	0.93
Ventricles (shape)	0.03	1.13	0.30	0.05	0.34	0.73	−0.13	−0.88	0.38	0.02	1.38	0.17	−0.01	−1.66	0.09	0.01	1.21	0.23

Regression results and predictive values of diagnosis, sex, age, ICV, and FIQ on asymmetry. Estimated regression coefficients reflect differences in ASD relative to controls. For diagnosis, negative beta values indicate that ASD>Controls, positive values indicate that ASD<Controls. For sex, negative beta values indicate that males>females, positive values indicate that males<females. All F(df1, df2) = 25, 922. Bold values indicate significance after Bonferroni correction.

**Fig. 3:**
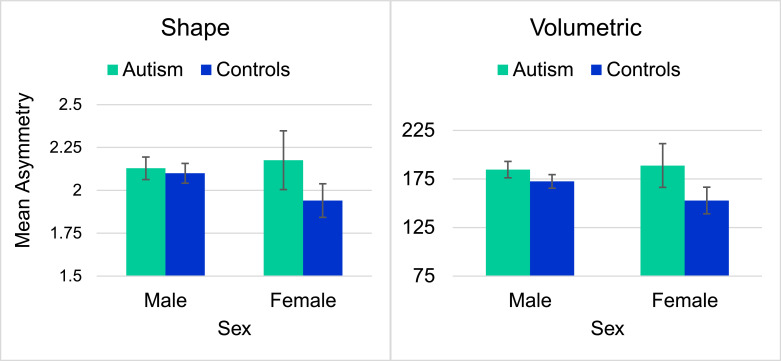
Mean amygdala asymmetry values by diagnosis and sex.

For the amygdala, diagnosis had no significant effect on shape or volumetric asymmetry, however, sex was a significant predictor of shape asymmetry. Irrespective of diagnosis, we observed significantly greater amygdalar shape asymmetry for males compared to females. Plots for the volumetric and shape asymmetry of the amygdala can be seen in Fig. 3. Although visual inspection of Fig. 3 appears to suggest that a diagnosis by sex interaction could be present, Welch's Two Sample T-Tests did not show any significant group differences in the amygdala for either measure of asymmetry.

**Fig. 4:**
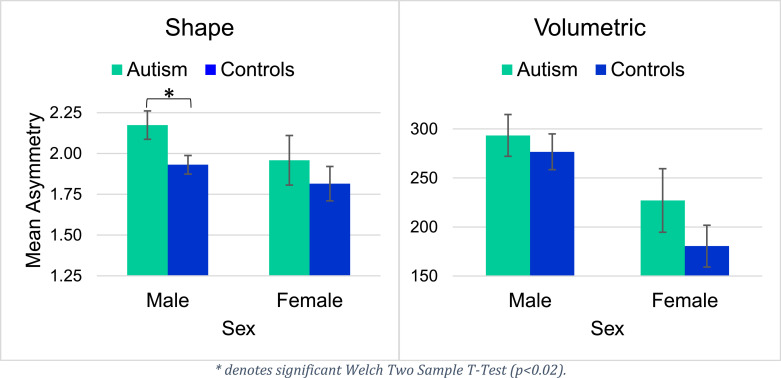
Mean hippocampal asymmetry values by diagnosis and sex.

For the hippocampus, diagnosis significantly predicted shape asymmetry, but did not explain volumetric hippocampal asymmetry. Sex was found to be a significant predictor for both volumetric and shape asymmetries in the hippocampus. We observed significantly greater shape asymmetry of the hippocampus in ASD compared to healthy controls, as well as a significantly greater shape and volumetric asymmetry of the hippocampus for males compared to females across both participant groups. In Supplementary Figure 1, we illustrate a scatter plot between the predicted and the measured asymmetry values. Welch's Two-Sample T-Tests revealed significant group differences in hippocampal shape asymmetry in males (*p* < 0.02), but not in females. Plots for volumetric and shape asymmetry of the hippocampus can be seen in Fig. 4.

**Fig. 5:**
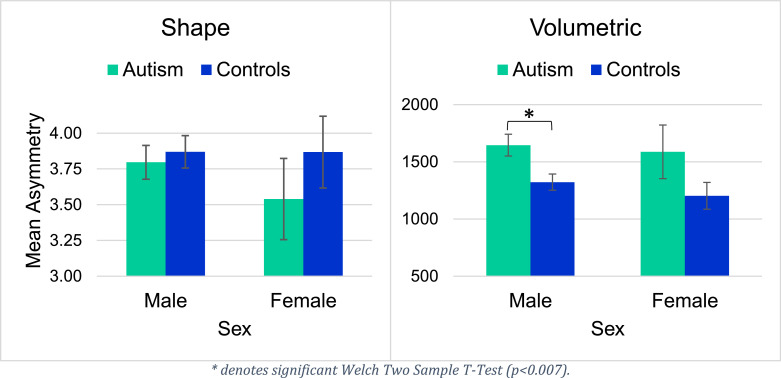
Mean ventricular asymmetry values by diagnosis and sex.

Notably, none of the regression factors explained ventricular shape asymmetry, but diagnosis did significantly predict volumetric ventricular asymmetry. We observed significantly greater volumetric ventricular asymmetry in individuals with ASD than in healthy controls. In Supplementary Figure 2, we illustrate a scatter plot between the predicted and the measured asymmetry values. Welch's Two-Sample T-Tests also revealed significant group differences in volumetric ventricular asymmetry in males (*p* < 0.007), though not in females. Fig. 5 displays mean ventricular asymmetry values.

Additionally, age was not associated with either type of asymmetry in the amygdala or the hippocampus, but age was a significant predictor for volumetric ventricular asymmetry (*p* = 0.03). As age increased, asymmetry within the ventricles increased, for both ASD patients and controls. The interaction between age and diagnosis was not significant for any of the three regions, however. Furthermore, the interaction between sex and age was not significant for any of the three regions.

**Fig. 6:**
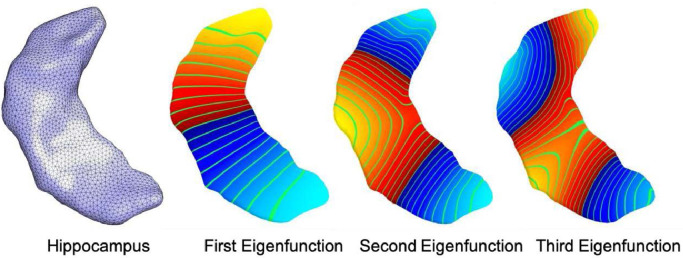
Visualization of the hippocampus mesh and the first three non-constant eigenfunctions of the Laplace-Beltrami operator calculated on the surface. Increasing positive values of the eigenfunctions are shown in the color gradient from red to yellow and decreasing negative values are shown from dark blue to light blue. Level sets are shown in green. (For interpretation of the references to color in this figure legend, the reader is referred to the web version of this article.)

As there are significant associations between hippocampal shape asymmetry and Autism, we illustrate the hippocampus mesh together with three non-constant eigenfunctions in Fig. 6. The eigenfunctions demonstrate natural vibrations of the shape when oscillating at a frequency specified by the square root of the eigenvalue. The main variation of the first eigenfunction is from top to bottom and the second one from left to right. The third one already shows a more complex pattern.

## 4 Discussion

The present study is the first to report volumetric and shape asymmetry results for the amygdala, hippocampus, and ventricles in a large cohort of individuals with ASD. We examined these differences in asymmetry in a large sample of ASD patients and typically developing healthy control subjects using linear regression analyses and a new measure of asymmetry based on spectral shape descriptors, introduced by Wachinger et al. (2016). While we did not find significant differences in shape or volume asymmetry of the amygdala in ASD patients, we did observe significantly increased shape asymmetry in the hippocampus, as well as increased volumetric asymmetry in the lateral ventricles in ASD patients.

Although we anticipated significant results for the amygdala, given its important role in impaired functions in ASD, diagnosis did not explain either measure of asymmetry in the amygdala. However, patients with ASD did have greater mean amygdalar asymmetry values than controls, for both measures of amygdalar asymmetry. Sex was found to be a significant predictor for shape asymmetry of the amygdala, but not for volumetric asymmetry, although males were found to have greater asymmetry than females for both measures of asymmetry in the amygdala. The absence of significant sex differences in volumetric amygdalar asymmetry is consistent with the recent report from Guadalupe et al. (2016), who found no significant differences in volumetric asymmetry of the amygdala between the sexes in more than 14,000 subjects, the largest sample to date.

Diagnosis was found to be a significant predictor for hippocampal shape asymmetry, however, diagnosis did not explain volumetric hippocampal asymmetry, despite that ASD patients did have higher mean volumetric hippocampal asymmetry values than controls. Sex was a significant predictor for both shape and volumetric asymmetries in the hippocampus, such that males had greater asymmetry than females, which is consistent with sex effects on volumetric hippocampal asymmetry previously described by Guadalupe et al. (2016). The opposite pattern of regression results was found for the ventricles—diagnosis explained volumetric asymmetry, but not shape asymmetry, and sex did not explain either measure of ventricular asymmetry. We argue that our differential findings for the hippocampus and lateral ventricles could simply be a result of structural differences of these two regions. Given the heterogeneous nature of the hippocampus and its substructures, compared to the more homogenous nature of the ventricles, this may account for the differential results of the volumetric and shape asymmetry analyses.

Since normalized eigenvalues were used for the shape descriptors, where the volume information is removed, it is possible that the shape descriptor does not detect purely volumetric differences that do not exert any influence on the shape of the structure. For the ventricles, which are fluid-filled structures and therefore intrinsically more homogeneous, it is reasonable to assume that significant changes in volume are more likely to occur than changes in the shape of the ventricles in the course of the disorder. In contrast, considering the heterogeneous composition of the hippocampus and its subfields, it is likely that focalized changes may occur within these subfields, which could result in detectable shape differences but not volumetric differences. Our hypothesis is consistent with previous findings of hippocampal shape abnormalities observed in ASD (Dager et al., 2007). An asymmetry in texture features of the hippocampus has been reported by Chaddad et al. (2017).

While Dager et al. (2007) did not specifically investigate asymmetry, they did find differences in hippocampal shape which distinguished children with ASD from typically developing children. They observed a pattern of upward bending of both the head and tail of the hippocampus, as well as an inward deformation of the subiculum in individuals with ASD, which is in line with our argument that focalized changes may occur in ASD, and that shape descriptors are sensitive enough to detect such focalized changes in subfields of the hippocampal formation. Given that our results showed hippocampal shape differences within a large cohort, hippocampal shape alterations may be worthy of consideration when developing MR-based classification algorithms and computer-aided diagnostic systems for ASD. Moreover, the supplementary role of shape analyses is certainly worthy of consideration, not only in the context of research on ASD, but for research on anatomical variations in general, given that such analyses permit the detection of focalized abnormalities that could otherwise remain undetected using purely volumetric measures.

It is important to note the methodological limitations of this study. Firstly, although FSIQ was controlled for, the ASD and control samples had an uneven number of subjects and were not matched with each other based on FSIQ, and FSIQ did differ significantly between the two groups. Additionally, given that the ASD sample was limited to relatively high-functioning individuals with ASD (FSIQ mean = 106.58, *SD* = 14.23), this may have had a significant influence on the results. Lower-functioning individuals with ASD could potentially exhibit significant structural abnormalities that differ from those found in high-functioning individuals with ASD. Thus, the generalizability of the current findings to the whole ASD spectrum is limited.

Second, several researchers have raised valid concerns and criticisms regarding the ABIDE dataset. Some of the most prevalent of these concerns include the confounding effects of scanner variations across ABIDE data collection sites (Auzias et al., 2015; Sato et al., 2016), the age range of subjects included in the dataset (Kazeminejad and Sotero, 2019), and significant differences in sample characterization variables, such as age and IQ across sites (Sato et al., 2016). However, although MRI data processing parameters as well as scanner parameters (e.g. scanner field strength, differing pule sequences) can increase variability and impact reliability of results (Han et al., 2006), some studies have demonstrated that site and scanner effects observed in multisite analyses may be minimal and outweighed by significant subject effects (Noble et al., 2017). Therefore, we believe that the use of ABIDE is still valid, however, future studies may benefit from analyzing these structures within additional datasets and employing cross-validation techniques.

Finally, our study also succumbs to the causal inference problem that often occurs with investigations of this nature. Although we observed differences in asymmetry among individuals with ASD and healthy controls, it is not clear whether ASD or the asymmetry causes the other. Longitudinal studies would provide stronger evidence in this regard, given that they would provide the opportunity to investigate the developmental trajectory of the disorder and its associated morphometric alterations. Future studies investigating longitudinal structural differences in individuals with ASD at varying levels of functioning and symptom severity are certainly warranted in order to better understand the relationship between symptom severity of ASD and structural patterns that emerge over time.

In summary, this study investigated volumetric and shape asymmetry in Autism Spectrum Disorder. We analyzed data from a large multi-site MRI dataset and found increased shape asymmetry of the hippocampus in individuals with ASD relative to controls. In addition, we observed increased volumetric asymmetry of the lateral ventricles in ASD. We believe that contralateral brain structures present a unique, within-patient reference element for disease progression. Therefore, asymmetries of contralateral structures could provide a personalized measure of the accumulation of past disease and disordered processes. The potential for these asymmetries and other previously reported structural abnormalities to serve as biomarkers for ASD warrants further studies utilizing large cohorts, especially longitudinal analyses and classification experiments, if this information is to be incorporated in a neurodiagnostic tool for ASD.

### Disclosures

No commercial support was received for the preparation of this manuscript and the authors have no conflicts of interest to report.

### Ethical approval

All procedures performed in studies involving human participants were in accordance with the ethical standards of the institutional and/or national research committee and with the 1964 Helsinki declaration and its later amendments or comparable ethical standards.

### Informed consent

Informed consent was obtained from all individual participants included in the original data sources on which this study is based.

## CRediT authorship contribution statement

**Rose Richards:** Conceptualization, Data curation, Formal analysis, Investigation, Resources, Writing - original draft, Writing - review & editing, Visualization, Project administration. **Ellen Greimel:** Conceptualization, Resources, Writing - review & editing. **Dorit Kliemann:** Writing - review & editing. **Inga K. Koerte:** Writing - review & editing. **Gerd Schulte-Körne:** Writing - review & editing, Supervision. **Martin Reuter:** Methodology, Resources, Writing - review & editing. **Christian Wachinger:** Conceptualization, Methodology, Software, Formal analysis, Investigation, Resources, Data curation, Writing - original draft, Writing - review & editing, Visualization, Supervision, Project administration, Funding acquisition.

## Appendix Supplementary materials

Image, application 1
