# Supplementary material for: Increased hippocampal shape asymmetry and volumetric ventricular asymmetry in autism spectrum disorder
Source: Neuroimage Clin. 2020 Feb 5;26:102207. doi: 10.1016/j.nicl.2020.102207 (PMC7037573; doi:10.1016/j.nicl.2020.102207)
Supplement: Supplementary file 1 [file mmc1.docx]

**Supplementary Material**

for

Increased Hippocampal Shape Asymmetry and
Volumetric Ventricular Asymmetry in Autism

Supplementary Figure 1: Scatter plot for the hippocampal shape asymmetry. On the x-axis are the predicted values from the regression model and on the y-axis are the measured asymmetry values.

Supplementary Figure 2: Scatter plot for the ventricular volume asymmetry. On the x-axis are the predicted values from the regression model and on the y-axis are the measured asymmetry values.
